# Perioperative routines and surgical techniques for saphenous vein harvesting in CABG surgery: a national cross-sectional study in Sweden

**DOI:** 10.1186/s13019-020-1056-y

**Published:** 2020-01-08

**Authors:** Hanna Larsson, Maria Hälleberg-Nyman, Örjan Friberg, Karin Falk-Brynhildsen

**Affiliations:** 1 Department of Cardiothoracic and Vascular Surgery, Faculty of Medicine and Health, Örebro University, Örebro, Sweden; 2 Faculty of Health and Medicine, School of Health Sciences, Örebro University, SE-701 82 Örebro, Sweden

**Keywords:** Perioperative hygiene routines, Surgical methods, Vein harvesting

## Abstract

**Background:**

The saphenous vein is the most commonly used conduit for coronary artery bypass grafting (CABG). Wound healing complications related to saphenous vein harvesting are common, with reported surgical site infection rates ranging from 2 to 20%. Patients’ risk factors, perioperative hygiene routines, and surgical technique play important roles in wound complications. Here we describe the perioperative routines and surgical methods of Swedish operating theatre (OT) nurses and cardiac surgeons.

**Methods:**

A national cross-sectional survey with descriptive design was conducted to evaluate perioperative hygiene routines and surgical methods associated with saphenous vein harvesting in CABG. A web-based questionnaire was sent to OT nurses and cardiac surgeons at all eight hospitals performing CABG surgery in Sweden.

**Results:**

Responses were received from all hospitals. The total response rate was 62/119 (52%) among OT nurses and 56/111 (50%) among surgeons.

Chlorhexidine 5 mg/mL in 70% ethanol was used at all eight hospitals. The OT nurses almost always (96.8%) performed the preoperative skin disinfection, usually for three to 5 minutes. Chlorhexidine was also commonly used before dressing the wound.

Conventional technique was used by 78.6% of the surgeons, “no-touch” by 30.4%, and both techniques by 9%. None of the surgeons used endoscopic vein harvesting. Type of suture and technique used for closing the wound differed markedly between the centres.

**Conclusions:**

In this article we present insights into the hygiene routines and surgical methods currently used by OT nurses and cardiac surgeons in Sweden. The results indicate both similarities and differences between the centres. Local traditions might be the most important factors in determining which procedures are employed in the OT. There is a lack of evidence-based hygiene routines and surgical methods.

## Background

The saphenous vein (SV) is the most commonly used conduit for coronary artery bypass grafting (CABG) [1, 2]. Wound healing complications related to saphenous vein harvesting (SVH) are common, with reported surgical site infection (SSI) rates ranging from 2 to 20% [3, 4]. Such complications are both a major cause of patient morbidity and an economic burden for the health care system [5]. Patient-dependent risk factors for SSI include diabetes, obesity, advanced age, female gender, and peripheral vascular disease [1, 3]. Several perioperative hygiene routines are aimed at maintaining an aseptic environment and avoiding endogenous or exogenous bacterial contamination: patient skin preparation and draping, routines for handling the surgical equipment, and postoperative dressing [6, 7].

The surgical techniques used for harvesting the SV and closing the wound also play an important role in wound complications [8, 9]. The open harvest technique, which uses a longitudinal skin incision over the vein, might facilitate the occurrence of wound complications. As an alternative to the open conventional technique (CT), the “no-touch” technique (NT), where the vein is harvested along with the perivascular adipose tissue was introduced at our department and published for the first time in 1996 [10]. When closing the wound, the number of subcutaneous suture layers as well as the choice of the suture material might influence the incidence of complications [11–13]. An endoscopic vein harvesting (EVH) technique can also be used in order to reduce the incidence of wound complications [8].

The perioperative hygiene routines and varieties in surgical methods for SVH are unfortunately often poorly evaluated scientifically. We therefore hypothesised that these routines might be based more on local traditions than on scientific evidence, and that surgical methods might differ substantially between different centres. To our knowledge, no previous studies have investigated current routines and surgical methods regarding SVH for CABG.

The aim of this nationwide study was to determine current practice in terms of perioperative hygiene routines and surgical methods used by Swedish OT nurses and cardiac surgeons regarding saphenous vein harvesting for CABG.

## Methods

### Study design and setting

The study had a descriptive design. A national web-based cross-sectional survey regarding perioperative routines and surgical methods associated with SVH for CABG was conducted in Sweden during February and March 2018.

### Questionnaires

Two study-specific self-report web questionnaires were constructed, one for OT nurses and one for surgeons, with questions concerning perioperative hygiene routines as well as surgical methods. (In Sweden, the OT nurse prepares the patient for the surgical procedure.)

The survey was sent to all OT nurses and surgeons, since it was not possible to select only those who performed CABG. The study-specific questionnaires were based on both existing research [1, 2, 8, 9, 11, 14–18] and the authors’ own clinical experience as OT nurses, a nurse, and a cardiothoracic surgeon.

The OT nurses were asked 32 open-ended questions regarding hygiene routines, dressing, and compression of the leg wound. The surgeons were asked 17 questions focusing on surgical technique regarding harvesting the SV, closing the wound, and type of suture. Questions regarding postoperative complications will be presented elsewhere.

Before the questionnaire was distributed, a pilot test was carried out among one OT nurse with context knowledge and one anaesthetic nurse with methodological knowledge at our institution, in order to test the content and its understanding. No corrections were needed after feedback from these two nurses. The OT nurse who pilot-tested the questionnaire was included in the study.

Reminders were sent after two and 4 weeks to those who had not yet answered the web questionnaire.

### Participants

The survey was sent to all surgeons and OT nurses at all eight hospitals where CABG surgery is performed in Sweden: Karlskrona Hospital, Karolinska University Hospital, Linköping University Hospital, Lund University Hospital, Sahlgrenska University Hospital, Umeå University Hospital, Uppsala University Hospital, and Örebro University Hospital. The surgeons were identified from the membership list of the Swedish Association for Cardiothoracic Surgery, including both consultant surgeons and residents. The OT nurses were identified by the head of the section at each hospital.

### Ethical considerations

The Regional Ethical Review Board of Uppsala, Sweden, advised that no formal ethical approval was required (reference number: 2017/519). The results from the questionnaire were confidential, and no associations were made between the results and any specific surgeon or OT nurse.

### Statistical analysis

Data were analysed using version 22 of the SPSS software package (SPSS 22 IBM, SPSS Inc., Chicago, USA).

## Results

Responses were received from all hospitals. Among the 119 OT nurses and 111 surgeons who were identified, the total response rate was 62/119 (52%) from the OT nurses and 56/111 (50%) from the surgeons.

### Perioperative routines

The OT nurses almost always (96.8%) performed the preoperative skin disinfection. Chlorhexidine 5 mg/mL in 70% ethanol was used in all cases. The skin disinfection was most commonly performed for three to 5 minutes. Incision film was seldom used on the legs. The instruments for harvesting the saphenous vein were separated from the ones used in the sternal wound by 93.5% of the OT nurses, and 75.8% used a separate table for the leg wound surgeon (Table 1).

**Table 1:** Operating theatre nurses’ preoperative routines regarding saphenous vein harvesting

Variable	OT nurses (*N* = 62)
Place of preparing equipment and surgical instruments, n (%)	
In the preparation room	11 (17.7)
In the OT	51 (82.3)
The sterile table is covered preoperatively, n (%)	
Yes	59 (95.2)
No	3 (4.8)
Time of preparing equipment and surgical instruments, n (%)	
Before the patient arrives in the OT	56 (90.3)
When the patient is being prepared in the OT	6 (9.7)
Separate table for instruments used in the vein harvesting, n (%)	
Yes	47 (75.8)
No	15 (24.2)
Separate instruments used in the vein harvesting, n (%)	
Yes	58 (93.5)
No	4 (6.5)
Skin disinfection, n (%)	
OT nurse	60 (96.8)
Surgeon	1 (1.6)
Assistant	1 (1.6)
Skin disinfection solution, n (%)	
Chlorhexidine 5 mg/ml in 70% ethanol	62 (100)
Ethanol 70%	0 (0)
Iodine	0 (0)
Chloraprep 2%	0 (0)
Estimated time for skin disinfection of leg, n (%)	
1–3 min	18 (29.0)
3–5 min	36 (58.1)
5–10 min	8 (12.9)
Tinted skin disinfection solution, n (%)^a^	
Yes	39 (62.9)
No	21 (33.9)
Skin disinfection of both legs, n (%)	
Yes	44 (71.0)
No	18 (29.0)
Leg lift for skin disinfection, n (%)	
Manually by staff	53 (85.5)
Leg lift device	9 (14.5)
Incision film on the leg/legs, n (%)	
Always/almost always	0 (0)
Always/almost always on any damaged skin on the leg	1 (1.6)
Sometimes	0 (0)
Never/almost never	61 (98.4)
Incision film with iodine, n (%)	
Yes	9 (14.5)
No	12 (19.4)
Never use incision film	41 (66.1)
Integuseal on the leg/legs, n (%)^a^	
Always/almost always	0 (0)
Sometimes	1 (1.6)
Never/almost never	58 (93.5)
Non-operated leg is covered, n (%)^a^	
Always/almost always	42 (67.7)
Sometimes	12 (19.4)
Never/almost never	6 (9.7)
Vein harvesting begins before the patient is totally draped for the heart surgery, n (%)^a^	
Always/almost always	10 (16.1)
Sometimes	10 (16.1)
Never/almost never	41 (66.1)

^a^Missing answer from OT nurse/nurses

Almost all OT nurses (98.4%) reported that the assistant surgeon changed gloves after SVH and before assisting in the sternal wound (Table 2). Cleaning the leg wound with chlorhexidine 5 mg/mL in 70% ethanol was common before dressing, and the majority of the nurses reported that they paid special attention to removing clots and blood from deep within the leg wound. Semipermeable dressings were used by 51.6% and nonpermeable dressings by 45.2% of the OT nurses. Most nurses applied an elastic bandage after the wound was dressed.

**Table 2:** Operating theatre nurses’ intraoperative and postoperative routines regarding saphenous vein harvesting

Variable	OT nurses (*N* = 62)
Gloves changed after vein harvesting, n (%)	
Always/almost always	61 (98.4)
Sometimes	1 (1.6)
Never/almost never	0 (0)
Elastic bandage used intraoperatively, n (%)^a^	
Always/almost always	59 (95.2)
Sometimes	1 (1.6)
Never/almost never	0 (0)
Position changes used to avoid pressure damage, n (%)^a^	
Always/almost always	2 (3.2)
Sometimes	16 (25.8)
Never/almost never	43 (69.4)
Bleeding control of the leg wound during ECC^b^, n (%)	
Yes	19 (30.6)
No	43 (69.4)
Suturing the leg/legs, n (%)	
Attending in cardiothoracic surgery	6 (9.7)
Resident in cardiothoracic surgery	13 (21.0)
Intern	29 (46.8)
OR nurse	4 (6.5)
Other	10 (16.0)
Drain to the leg, n (%)	
Always/almost always	0 (0)
Sometimes	11 (17.7)
Never/almost never	51 (82.3)
Clots and blood removed before dressing, n (%)	
Always/almost always	59 (95.2)
Sometimes	3 (4.8)
Never/almost never	0 (0)
Cleaning solution before dressing, n (%)	
Sodium chloride	2 (3,2)
Chlorhexidine 5 mg/ml in 70% ethanol	43 (69.4)
Other chlorhexidine solutions	17 (27.4)
Semipermeable dressing on the leg/legs, n (%)^a^	
Always/almost always	32 (51.6)
Sometimes	0 (0)
Never/almost never	26 (41.9)
Nonpermeable dressing on the leg/legs, n (%)^a^	
Always/almost always	28 (45.2)
Sometimes	3 (4.8)
Never/almost never	28 (45.2)
Dressing application on the leg/legs, n (%)	
Attending in cardiothoracic surgery	1 (1.6)
Resident in cardiothoracic surgery	3 (4.8)
Intern	7 (11.3)
OR nurse	50 (80.6)
Other	1 (1.6)
Elastic bandage used after the leg wound is dressed, n (%)^a^	
Always/almost always	56 (90.3)
Sometimes	3 (4.8)
Never/almost never	2 (3.2)
Compression stocking used after the leg wound is dressed, n (%)^a^	
Always/almost always	11 (17.7)
Sometimes	0 (0)
Never/almost never	50 (80.6)
Leg compression, n (%)^a^	
Lower limb	20 (32.3)
Whole leg	41 (66.1)
No compression	0 (0)

^a^Missing answer from OT nurse/nurses

^b^*ECC* Extracorporeal Circulation

### Surgical methods

Data on the surgeons’ vein harvest techniques are shown in Table 3. The majority of the surgeons used CT for SVH, while 30.4% used NT. Some of the respondents used both techniques. Notably, none in this study used EVH.

**Table 3:** Cardiac surgeons’ routines regarding saphenous vein harvesting

Variable	Cardiothoracic surgeon (*N* = 56)
Own frequency of SVH, n (%)	
> 20 times/year	32 (57.1)
11–20 times/year	12 (21.4)
6–10 times/year	5 (8.9)
1–5 times/year	5 (8.9)
Never	2 (3.6)
Vein harvester, n (%)	
Attending in cardiothoracic surgery	17 (30.4)
Resident in cardiothoracic surgery	16 (28.6)
Operating theatre nurse	5 (8.9)
Intern	14 (25.0)
Operations assistant	1 (1.8)
Vein harvester	2 (3.6)
Common clinical technique for SVH, n (%)	
Yes	12 (21.4)
Partly	35 (62.5)
No	7 (12.5)
Type of vein graft, n (%)^a^	
Conventional	44 (78.6)
“No-touch”	17 (30.4)
Endoscopic	0 (0)
Ligation of side branches, n (%) ^a^	
Clips	34 (60.7)
Absorbable ligature	18 (32.1)
Non-absorbable ligature	27 (48.2)
Other	1 (1.8)
Diathermy/coagulation, n (%)	
Monopolar diathermy	40 (71.4)
Bipolar diathermy	10 (17.9)
Ultrasound “harmonic scalpel”	1 (1.8)
Never use diathermy	3 (5.4)
Suturing the leg/legs, n (%)	
Attending in cardiothoracic surgery	13 (23.2)
Resident in cardiothoracic surgery	11 (19.6)
Operating room nurse	4 (7.1)
Intern	23 (41.1)
Operations assistant	1 (1.8)
Vein harvester	2 (3.6)
Skin suture, n (%)	
Intracutaneous suture	54 (96.4)
Skin staples	0 (0)
Suture layers in subcutis, n (%)	
One layer	23 (41.1)
Two layer	9 (16.1)
No subcutaneous layer	22 (39.3)
Subcutaneous suture, n (%)	
Braided	23 (41.1)
Monofilament	24 (42.9)
No subcutaneous suture	9 (16.1)
Intracutaneous suture, n (%)	
Braided	4 (7.1)
Monofilament	50 (89.3)
Suture with triclosan (Vicryl+), n (%)	
Always	23 (41.1)
Sometimes	9 (16.1)
Never	22 (39.3)

^a^n is more than 56 because it was possible to give more than one answer

The use of no or only one subcutaneous suture layer was most common, but 16.1% used two layers. There was a marked variation in the use of braided or monofilament subcutaneous sutures as well as of triclosan-containing sutures, with equal proportions of surgeons reporting that they never or always used them. All respondents in the study sutured the wound intracutaneously; none used skin staplers.

## Discussion

This study is the first survey describing perioperative hygiene routines and surgical methods regarding SVH in Sweden. The main finding was that although perioperative hygiene routines were similar between the centres and the interventions recommended by the national guidelines were implemented in daily work, aspects such as the techniques for harvesting the vein and closing the wound differed markedly between the surgeons and centres.

There are differences internationally regarding preoperative preparation of the surgical patient. Depending on country, the patient may be prepared in the OT by the surgeon or by nurses with differing educational levels and training. In Sweden, the OT nurse has an independent role and takes responsibility for OT aseptic techniques, skin preparation and draping of the patient, and generally maintaining good care for the surgical patient, such as preventing hypothermia and decubitus [7, 19].

Chlorhexidine 5 mg/mL in 70% ethanol was used in all centres, and the skin disinfection was most commonly performed for three to 5 minutes according to national guidelines [20]. Almost all of the OT nurses had a separate table and/or separate instruments for the vein harvesting, a standard routine which probably stems from a general belief that bacteria can spread from the leg wound to the sternotomy. However, in contradiction of this belief, we showed in a previous study that there is almost no intraoperative bacterial growth on the skin or in the subcutaneous tissue at the harvest site on the leg [18]. In contrast, there is bacterial growth subcutaneously in the sternotomy during surgery in most patients [21]. Thus, in our opinion, the generally accepted routine in which the assistant surgeon changes gloves after vein harvesting lacks evidence and is not justified. On the other hand, studies have shown that the micro perforation rate of gloves increases over time, and bacteria could possibly pass through the perforation [22–24] and contaminate the wound. There are no Swedish guidelines for when surgical gloves should be changed, but international guidelines from the Association of Perioperative Registered Nurses [25] recommend changing the outer glove every 90 min whether the gloves are macroscopically damaged or not. It could be relevant to apply these guidelines also in Sweden.

The harvest technique has a contributory role in the development of leg wound SSI. The open harvest approach increases the risk for SSI compared with the EVH technique [8, 9, 15]. However, some studies report that the EVH technique might impair graft patency [8, 26]. This might be the main reason why, in this study, the open vein harvesting technique was used by 100% of respondents. The NT has gradually increased in popularity [27], possibly because graft patency with this technique have been demonstrated to be superior to those with CT [17, 28]. Nevertheless, CT was used by 78.6% of the participants in the present study. It remains to be seen whether the choice of CT or NT for open vein harvesting technique significantly influences the rate of leg wound SSI, but results from at least one ongoing large multicentre randomised controlled trial comparing the two techniques (NCT03501303) should provide more information.

Our participants differed in terms of the subcutaneous suture layer and types of sutures used to close the leg wound. One study found that the subcutaneous suture did not reduce the postoperative infection rate [12], while another showed that wound gaps were associated with SSI in the leg [11]. The latter study [11] also showed that the choice of suture and suturing technique is important to prevent SSI in the leg. A lower rate of SSI has been found in cases where the assistant surgeon was experienced [12]. Approximately half of the surgeons in the present study used sutures coated with triclosan. Prospective randomised studies have yielded differing results [13, 29]. In one study of 328 CABG patients, triclosan-coated sutures did not reduce SSI in the leg wound [29]. However, in a later randomised controlled trial of 374 patients, the SSI rate was significantly reduced with triclosan sutures [13].

The high incidence of SSI in prospective studies is a pertinent and troublesome issue, and many factors affect the results. Evidence is lacking, and there are a number of routines and methods which are not based on evidence but have not been de-implemented. One routine lacking evidence is the routine of preparing a separate table and/or separate instruments for the vein harvesting, mentioned above. De-implementation is a process for identifying and removing practices based on tradition and habits which lack adequate scientific support [30]. In the pursuit of evidence-based health care, de-implementation of old routines is just as important as the implementation of new evidence. The difficulties and success factors for introducing evidence-based practice in clinical work have been described in implementation research [31]. It is of the greatest importance to discuss and prepare implementation strategies for clinical practice [32].

This national cross-sectional survey was performed in order to identify unjustifiable differences or peculiarities in current practice which might signal a lack of scientific evidence. Our findings could provide a basis for further studies aimed at reducing the incidence of complications after SVH.

## Limitation

One limitation of the study was the self-designed questionnaires, which were used since we found no relevant existing survey material. This carried a risk that essential questions might be missed. To increase the content validity, the questionnaires were tested within a small pilot group before they were distributed to the full group. The response rates for the two questionnaires were 52% for the OT nurses and 50% for the surgeons, which may have led to response bias. We could also have sent a postal survey instead of a web-based questionnaire to allow for answering in different ways [33]. We believe that the response rate gives the study moderate external validity. A strength of the study was that there were respondents from all centres in Sweden. No comparisons between centres or respondents were performed.

## Conclusion

In this study we present an insight into the hygiene routines and surgical methods currently used by OT nurses and cardiac surgeons in Sweden. The results indicate both similarities and differences between the centres. There is a lack of evidence regarding common routines and methods. Local tradition might be one of the most important factor determining which procedures are employed in the OT.

## Data Availability

The datasets used and/or analysed during the current study are available from the corresponding author on reasonable request.

## Abbreviations

CABG: Coronary artery bypass grafting
CT: Conventional technique
EVH: Endoscopic vein harvesting
NT: “No-touch” technique
OT: Operating theatre
SSI: Surgical site infection
SV: Saphenous vein
SVH: Saphenous vein harvesting
